# Protective Effects of Hydrogen-Rich Saline on Rats with Smoke Inhalation Injury

**DOI:** 10.1155/2015/106836

**Published:** 2015-05-21

**Authors:** Xing Chen, Qi Liu, Dawei Wang, Shihai Feng, Yongjian Zhao, Yun Shi, Qun Liu

**Affiliations:** ^1^The Fourth Hospital of Tianjin, Tianjin 300222, China; ^2^Tianjin Medical University, Tianjin 300070, China

## Abstract

*Objective*. To explore the protective effects of hydrogen-rich saline on rats with smoke inhalation injury.* Methods*. 36 healthy male Sprague-Dawley rats were randomly divided into 3 groups (*n* = 12 per group): sham group (S), inhalation injury plus normal saline treatment group (I+NS), and inhalation injury plus hydrogen-rich saline treatment group (I+HS). 30 min after injury, normal saline and hydrogen-rich saline were injected intraperitoneally (5 mL/kg) in I+NS group and I+HS group, respectively. All rats were euthanized and blood and organ specimens were collected for determination 24 h after inhalation injury.* Results*. Tumor necrosis factor-alpha (TNF-*α*) levels, malondialdehyde (MDA) concentrations, nuclear factor kappa B (NF-*κ*B) p65 expression, and apoptosis index (AI) in I+HS group were significantly decreased (*P* < 0.05), while superoxide dismutase (SOD) activities were increased compared with those in I+NS group; and a marked improvement in alveolar structure was also found after hydrogen-rich saline treatment.* Conclusions*. Hydrogen-rich saline treatment exerts protective effects in acute lung injury induced by inhalation injury, at least in part through the activation of anti-inflammatory and antioxidant pathways and inhibition of apoptosis.

## 1. Introduction

Inhalation injury, when accompanied by multiple organ injury, is caused by heat and (or) smoke respiratory damage and contributes to being the leading cause of death in intensive care units, with a mortality that has remained in the range of 10%–20% [1].

Airway damage is an important pathological change in the process of inhalation injury. Due to the physiological structure, severe airway damage is mostly caused by smoke inhalation injury. The chemicals in the smoke can cause bronchitis and lead to the airway mucosa membrane hyperemia, edema, and hemorrhage and even mucosal erosion, necrotic damage, and ulceration and eventually result in dysfunctional ventilation function in the lung.

The release of oxygen free radicals, inflammation, and stress are important factors in inhalation injury. In the early stages of inhalation injury, the release of oxygen free radicals can directly cause the damage of the airway epithelium [2] and vascular endothelium by acting on the capillaries [3]. Plenty of evidence shows that the toxicity in the smoke can activate oxygen free radicals in lung tissue [4] and increase production of both cyclooxygenase and lipoxygenase products [5], which obviously increase microvascular permeability [6]. In addition, inflammation and stress have been implicated in the pathogenesis of secondary pulmonary injury, including activation of polymorphonuclear leukocytes which lead to damage of lung parenchyma [7].

Since Ohsawa et al. [8] found that animals which inhaled small doses of hydrogen (2%, 35 min) can significantly improve cerebral ischemia-reperfusion injury, hydrogen, as a nontoxic gas, has been proved to be a novel antioxidant through its selective reduction of the hydroxyl radical (^•^OH) and peroxynitrite (ONOO^−^), which are the most cytotoxic of reactive oxygen species (ROS) and reactive nitrogen species (RNS), while maintaining the metabolic oxidation-reduction reaction and other ROS, such as superoxide anion (O^2−^) and H_2_O_2_ which are less cytotoxic [9]. In recent years, a number of studies also found hydrogen had a good effect on anti-inflammatory and antiapoptotic regulation of signal transduction pathways besides selective antioxidant effects [10–13].

Considering the risk of explosion associated with H_2_ + air mixtures > 4.6% (v/v) as well as the safety and convenience of administration, this experiment was designed to investigate the protective effect of hydrogen-rich saline on acute lung injury (ALI) induced by inhalation injury.

## 2. Materials and Methods

### 2.1. Animals

Male Sprague–Dawley rats, weighing approximately 180 to 200 g, were purchased from the Experimental Animal Center, Tianjin Medical University (Tianjin, China). Those rats were housed in individual cages at 20°C to 22°C with a 12 h light/dark cycle and free access to food and water. All animals were housed and handled according to Tianjin University Institutional Animal Care and Use Committee guidelines and all animal work was approved by the appropriate committee (no. TMUaMEC2013006).

### 2.2. Experimental Design

36 healthy male Sprague-Dawley rats were randomly divided into 3 groups (*n* = 12 per group): sham group (S), inhalation injury plus normal saline treatment group (I+NS), and inhalation injury plus hydrogen-rich saline treatment group (I+HS). A standardized model of inhalation injury described by Xie et al. [14] was used with revision. Briefly, we placed smoking material into the combustion chamber, connected the smoke generator with the traumatogenic chamber, and maintained a CO concentration of 600 ppm in the traumatogenic chamber. The rats were anaesthetized with 2% pentobarbital sodium (50 mg/kg) administered intraperitoneally. Each rat was confined in the sealed traumatogenic chamber, inhaling smoke for 2 min. The rat was taken out of the chamber in order to breathe fresh air for 5 min before being reexposed to smoke for another 2 min. The above procedure led to severe inhalation injury of the rats but no death. Then, the procedure was repeated three times, following the traumatogenic pattern 2-5-2-5-2 (min). 30 min after injury, normal saline and hydrogen-rich saline were injected intraperitoneally (5 mL/kg) in I+NS group and I+HS group, respectively. All rats were euthanized and blood and organ specimens were collected for determination 24 h after inhalation injury.

### 2.3. Preparation of HS

Hydrogen gas was dissolved in normal saline for 6 h under high pressure (0.4 MPa) to a supersaturated level. The saturated HS was stored under atmospheric pressure at 4°C in an aluminum bag with no dead volume and was sterilized by *γ*-radiation. Determined by gas chromatography, hydrogen concentration in saline maintained at a concentration of 0.79 mmol/L. Hydrogen-rich saline was freshly prepared every week to ensure that the concentration of hydrogen was more than 0.6 mmol/L [15], which was in saturated state.

### 2.4. MDA and SOD Activity Assay

The left lower lobe lung samples were homogenized in chilled phosphate-buffered saline (PBS); the homogenate was then centrifuged at 5000 rpm for 10 min at 4°C. The supernatant was used to determine MDA and SOD. MDA assay reagents and SOD assay kits were purchased from the Nanjing Jiancheng Bioengineering Institute (Nanjing, China). MDA concentrations were determined by the thiobarbituric acid method and SOD activities were evaluated by the xanthine oxidase method. The absorbance was measured at 532 and 550 nm for MDA and SOD, respectively, with a spectrometer.

### 2.5. TNF-*α* Assay

The serums previously obtained were used for detecting the level of TNF-*α* by specific enzyme-linked immunosorbent assay kits (R&D Systems China Co. Ltd) using a microplate reader (CA 94089; Molecular Devices, Sunnyvale, Canada) according to the manufacturer's instructions.

### 2.6. Lung Histological Examination

The right lower lobe lung samples were fixed in 4% paraformaldehyde, embedded in paraffin, and sectioned to a thickness of 4 to 6 *μ*m. After deparaffinization and rehydration, the sections were stained with hematoxylin and eosin for light microscopy. Lung tissue pathological changes were observed and also evaluated morphologically by scoring histology specimens by two pathologists in a blind test. Edema, hyperemia and congestion, neutrophil margination and tissue infiltration, intra-alveolar hemorrhage and debris, and cellular hyperplasia were scored [16] as follows: 0 = absent, 1 = mild, 2 = moderate, and 3 = severe. A total score was calculated for each animal.

### 2.7. NF-*κ*Bp65 Expression

Immunohistochemistry was used to determine NF-*κ*B nuclear translocation of lung tissue sections. The assay was performed according to the manufacturer's instructions (Boster Inc. Wuhan, China). The primary antibody is NF-*κ*Bp65 polyclonal antibodies and the secondary antibody is sheep anti-rabbit IgG. Apoptotic cells were manifested by brownish staining in the nuclei and cytoplasm. Ten images were randomly selected from each section, and the apoptotic index was expressed as mean integrated optical density (MOD) [17]. Two pathologists performed a blind examination.

### 2.8. Detection of Apoptotic Cells

Terminal deoxynucleotidyl transferase dUTP nick end labeling (TUNEL) assay was used to monitor the extent of DNA fragmentation as a measure of apoptosis in paraffin-embedded sections. The assay was performed according to the manufacturer's instructions (Roche Diagnostics GmbH, Mannheim, Germany). Apoptotic cells manifested themselves in brownish staining in the nuclei. Ten images were randomly selected from each section for counting at least 1,000 cells, and the apoptotic index was expressed as a percentage of TUNEL-positive cells [17]. Two pathologists performed the blind examination.

### 2.9. Transmission Electron Microscopy

The middle lobes of the right lung were cut into 1 to 2 mm cubes and fixed in 2.5% glutaraldehyde at 4°C immediately. They were then treated with osmium tetroxide and dehydrated in serial concentrations of ethanol. The tissues were treated with propylene oxide again and then a propylene oxide/epoxy resin mixture. Finally they were embedded in labeled capsules with freshly prepared resin by Epon812. Sections were stained with uranyl acetate and lead citrate and observed with a Hitachi-7500 transmission electron microscope (Hitachi, Tokyo, Japan) at 10000 to 20000x magnifications.

### 2.10. Statistical Analysis

The measurement data were expressed as mean ± SD. Differences between groups were determined with one-way ANOVA followed by Student-Newman-Keuls test. The statistical analysis was performed with SPSS 16.0 software. In all tests, a value of *P* < 0.05 was considered statistically significant.

## 3. Results

### 3.1. MDA and SOD in Lung Tissue

**Figure 1:**
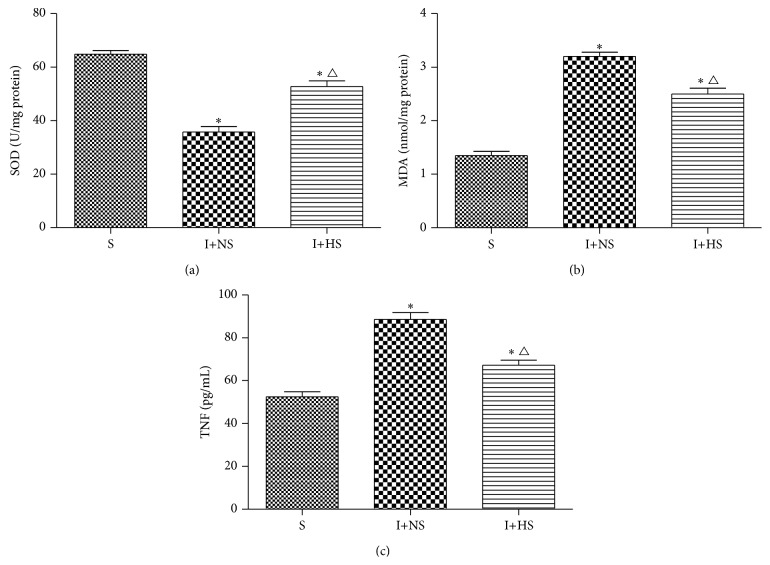
Hydrogen treatment upregulated the activities of lung antioxidant enzymes and reduced the levels of lung oxidative product and inflammatory cytokine in rats induced by inhalation injury. (a) Lung SOD activity. (b) Lung MDA level. (c) Serum TNF-*α* level. 30 min after injury, normal saline and hydrogen-rich saline were injected intraperitoneally (5 mL/kg) in I+NS group and I+HS group, respectively. The serum and tissue were harvested for measuring these indicators at 24 h after inhalation injury or sham injury. The values are expressed as mean ± SD (*n* = 12 per group). ^*∗*^ *P* < 0.05 versus S group; ^△^ *P* < 0.05 versus I+NS group.

As shown in Figure 1, rats in I+NS group demonstrated having significant change at 24 h after inhalation injury, which was assessed by lung MDA and SOD activity. Rats in I+NS group showed a significant increase in lung MDA activity and a significant decrease in SOD activity compared with those in S group (*P* < 0.01). These abnormal changes were significantly attenuated by hydrogen-rich saline treatment (Figures 1(a) and 1(b)).

### 3.2. TNF-*α* in Serum

TNF-*α* levels in I+NS group increased significantly compared with the S group (*P* < 0.01). TNF-*α* levels in I+HS group decreased significantly compared with the I+NS group (*P* < 0.05) (Figure 1(c)).

### 3.3. Lung Histological Examination

**Figure 2:**
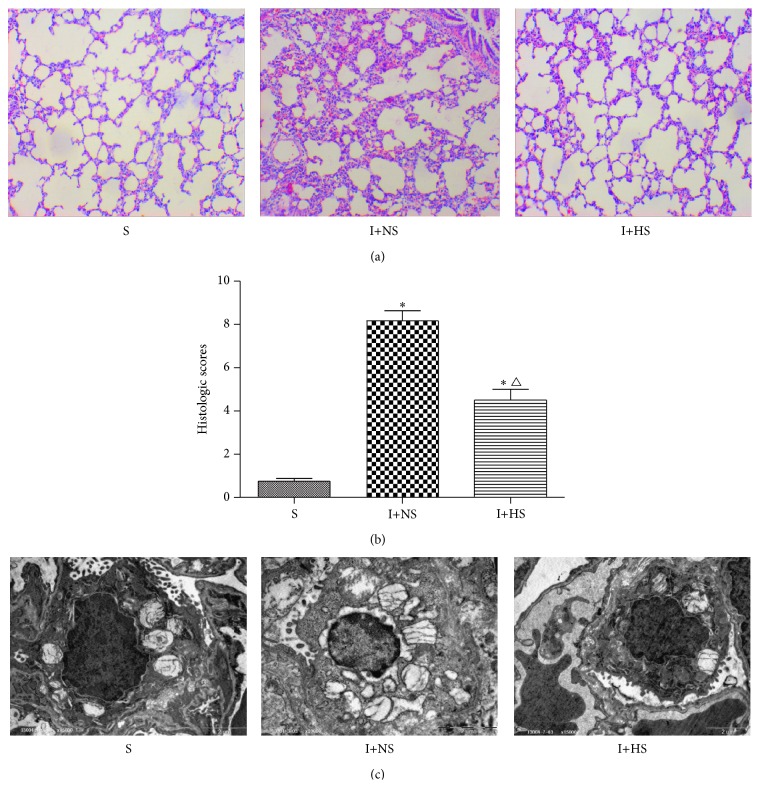
Hydrogen-rich saline treatment attenuated ALI in rats with inhalation injury. (a) Lung histopathologic change. (b) Lung histological scores. (c) Lung ultrastructural histopathologic change. The values are expressed as mean ± SD (*n* = 12 per group). ^*∗*^ *P* < 0.05 versus S group; ^△^ *P* < 0.05 versus I+NS group.

With respect to histopathologic changes observed in lung tissue, this study demonstrated that lung injury characterized by alveolar wall thickening, infiltration of neutrophils into the lung interstitium and alveolar space, consolidation, and alveolar hemorrhage was present in I+NS group. Hydrogen-rich saline treatment resulted in a reduction of infiltrated inflammatory cells and a remarkable improvement in lung architecture when compared with those in the I+NS group (Figure 2(a)). Lung histological scores in I+NS group increased significantly compared with the S group (*P* < 0.01). Lung histological scores in I+HS group decreased significantly compared with the I+NS group (*P* < 0.05) (Figure 2(b)).

### 3.4. Effects of Hydrogen-Rich Saline on NF-*κ*Bp65 Expression

**Figure 3:**
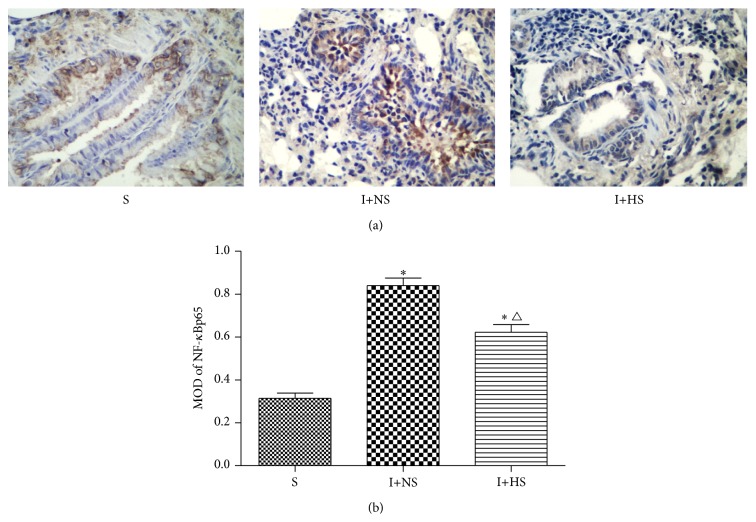
Hydrogen treatment inhibited the lung NF-*κ*Bp65 translocation and ameliorated lung injury. (a) Immunohistochemistry of NF-*κ*Bp65 positive cells of lung tissues (original magnification ×40). (b) MOD of NF-*κ*Bp65 positive cells. The values are expressed as mean ± SD (*n* = 12 per group). ^*∗*^ *P* < 0.05 versus S group; ^△^ *P* < 0.05 versus I+NS group.

Few positive staining cells of expression of NF-*κ*Bp65 were present in the lung tissue of S group, which were mainly located in the cytoplasm. NF-*κ*B positive cells in I+NS group were deeply stained and mainly located in nucleus, while they were significantly reduced in I+HS group. The statistical results of mean optical density (MOD) value for each group are consistent with the above statement; and the MOD values of I+HS group are significantly lower than I+NS group (*P* < 0.05) (Figure 3). As shown in Figure 3(a), NF-*κ*Bp65 were present mostly in the cytoplasm, with minimal localization in the nucleus. Inhalation injury produced a rapid translocation of NF-*κ*Bp65 into the nucleus. The nuclear translocation was accompanied by a corresponding decrease in NF-*κ*Bp65 in the cytoplasm; and hydrogen treatment could inhibit the lung NF-*κ*Bp65 translocation.

### 3.5. Effects of Hydrogen-Rich Saline on Cell Apoptosis

**Figure 4:**
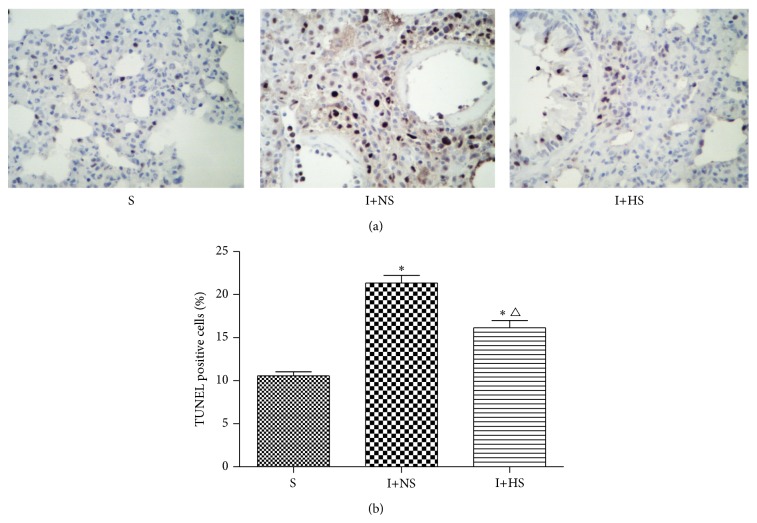
Hydrogen treatment prevented the lung cell apoptosis in rats with inhalation injury. (a) Lung TUNEL staining (original magnification ×40). (b) Percentage of TUNEL-positive cells. The values are expressed as mean ± SD (*n* = 12 per group). ^*∗*^ *P* < 0.05 versus S group; ^△^ *P* < 0.05 versus I+NS group.

Few cells were stained brown in S group, while plenty of positive cells were stained brown in I+NS group. Positive cells in I+HS group were significantly reduced compared with those in I+NS group. The statistical results of apoptotic index (AI) for each group are consistent with the above statement (Figure 4). These findings indicate that hydrogen treatment prevents the lung cell apoptosis in rats with inhalation injury.

### 3.6. Ultrastructural Organization of Lung Tissues

With respect to ultrastructural histopathologic changes under electron microscopy, Type I alveolar cells are characterized by vacuolation, degranulation, swelling of mitochondria, and dilation of perinuclear cisterna and rough endoplasmic reticulum pool after inhalation injury, while Type II alveolar epithelial cells are typically characterized by vacuolation of lamellar bodies and exfoliated tubular myelin. A marked improvement in alveolar structure was found after hydrogen-rich saline treatment when compared with I+NS group (Figure 2(c)).

## 4. Discussion

Our study demonstrated that hydrogen-rich saline treatment significantly ameliorated lung injury caused by inhalation injury in rats. Intraperitoneal administration of hydrogen-rich saline effectively diminished the lung damage induced by inhalation injury, attenuated the induction of proinflammatory cytokines into the lung tissue, increased activities of antioxidant enzymes, decreased the level of lipid peroxidation, and was associated with inhibition of apoptosis.

Oxygen free radicals and inflammatory response are known to play critical roles in the pathogenesis of smoke inhalation injury. After smoke inhalation injury, neutrophils, macrophages, and alveolar capillary endothelial cells release a variety of inflammatory mediators including reactive oxygen species (ROS) and reactive nitrogen species (RNS), free radicals, and many cytokines, such as TNF-*α* and IL-1 [18]. It results in a severe inflammatory response, which leads to priming and activation of leucocytes, destruction of pulmonary endothelium, extravasation of protein-rich fluid into the interstitium, and formation of edema. TNF-*α*, a potent proinflammatory and immunomodulatory cytokine, is well documented as a critical cytokine in the development of fibrosis [19]. The increased levels of TNF-*α* lead to increased production and activation of other proinflammatory cytokines and factors, such as IL-1, IL-6, and IL-8. Our experimental results show that the TNF-*α* level in I+HS group was significantly less than that in I+NS group, which demonstrates that hydrogen-rich saline treatment can reduce the degree of lung injury of inhalation injury via attenuating oxygen free radicals and inflammatory response. The mechanism of hydrogen-rich saline treatment may be to inhibit formation of oxygen free radicals and lessen oxidative damage by chelated iron in serum and brain and maintaining a certain level of serum copper [20].

Increased mucus secretion is one of the important characteristics of the response to smoke inhalation injuries [21]. Oxidative stress plays a key role in airway abnormal mucus production [22]. MDA, which is the end product of oxidative injury and a marker of lipid peroxidation, can indirectly reflect the severity of body cells attacked by oxygen free radicals, while SOD can indirectly reflect the ability of the body to eliminate oxygen free radicals [23]. In this study, MDA rapidly increased while SOD markedly decreased in lung tissues after smoke inhalation injury when compared with sham injury animals. We also observed that the MDA levels decreased significantly after hydrogen-rich saline treatment, while SOD levels were increased significantly in rats injured by smoke inhalation. The results suggest that the abated production of lung oxidative products and the improved endogenous antioxidant potential may attribute to the protection of HS treatment. Furthermore, our results of MDA levels in lung tissues are well consistent with the results of those in BALF after hydrogen-rich saline treatment [22].

A growing number of studies have found that NF-*κ*B, an important intracellular nuclear transcription factor, regulates the expression of various genes involved in inflammation and cytoprotection [24]. Several findings confirm that NF-*κ*B exists in an inactive state in the cytoplasm in unstimulated cells. However, after smoke inhalation injury, NF-*κ*B translocates to the nucleus and activates the expression of its target genes [25]. In the present study, we observed the increased expression of NF-*κ*Bp65 in the lung after smoke inhalation injury, while HS treatment significantly decelerated this trend. The results suggest that hydrogen-rich saline can ameliorate lung injury partly via inhibiting the activation of the NF-*κ*B to regulate the expression of the related gene.

A series of research studies confirm that apoptosis plays a major key role in the early stage of inhalation injury, which is involved in mitochondrial pathway [26]. Mitochondria is an important organelle which can regulate apoptosis by disrupting electron transport, oxidative phosphorylation, and adenosine triphosphate production, releasing proteins that trigger the activation of caspase family proteases and altering cellular reduction-oxidation potential [27]. In the present study, we found that the apoptotic cells of lung tissue in I+HS group were significantly less than those in I+NS group, which suggests that HS treatment can significantly reduce the number of apoptotic cells. In addition, we further found the abnormal changes of Type I and Type II alveolar cells described as above after inhalation injury and a marked improvement in alveolar structure after hydrogen-rich saline treatment.

## 5. Conclusions

In conclusion, the results of this study demonstrate that hydrogen-rich saline treatment exerts protective effects in acute lung injury induced by inhalation injury, at least in part through the activation of anti-inflammatory and antioxidant pathways and the inhibition of apoptosis. Although the exact mechanism involved in the protective role of HS remains to be determined, due to its safety, efficacy, and convenience, hydrogen-rich saline treatment should be considered as a potential therapy for acute lung injury induced by inhalation injury.
